# Oncogenic N-Ras Stimulates SRF-Mediated Transactivation via H3 Acetylation at Lysine 9

**DOI:** 10.1155/2018/5473725

**Published:** 2018-01-03

**Authors:** Sun-Ju Yi, Seong Yun Hwang, Myung-Ju Oh, Yang-Hoon Kim, Hojin Ryu, Sung-Keun Rhee, Byung H. Jhun, Kyunghwan Kim

**Affiliations:** ^1^School of Biological Sciences, College of Natural Sciences, Chungbuk National University, Cheongju, Chungbuk 361-763, Republic of Korea; ^2^Hazardous Substances Analysis Division, Seoul Regional Food and Drug Administration, Ministry of Food and Drug Safety, Seoul 07978, Republic of Korea; ^3^Department of Cogno-Mechatronics Engineering, Pusan National University, Busan 46241, Republic of Korea

## Abstract

Signal transduction pathways regulate the gene expression by altering chromatin dynamics in response to mitogens. Ras proteins are key regulators linking extracellular stimuli to a diverse range of biological responses associated with gene regulation. In mammals, the three ras genes encode four Ras protein isoforms: H-Ras, K-Ras4A, K-Ras4B, and N-Ras. Although emerging evidence suggests that Ras isoforms differentially regulate gene expressions and are functionally nonredundant, the mechanisms underlying Ras specificity and Ras signaling effects on gene expression remain unclear. Here, we show that oncogenic N-Ras acts as the most potent regulator of SRF-, NF-*κ*B-, and AP-1-dependent transcription. N-Ras-RGL2 axis is a distinct signaling pathway for SRF target gene expression such as Egr1 and JunB, as RGL2 Ras binding domain (RBD) significantly impaired oncogenic N-Ras-induced SRE activation. By monitoring the effect of Ras isoforms upon the change of global histone modifications in oncogenic Ras-overexpressed cells, we discovered that oncogenic N-Ras elevates H3K9ac/H3K23ac levels globally in the chromatin context. Importantly, chromatin immunoprecipitation (ChIP) assays revealed that H3K9ac is significantly enriched at the promoter and coding regions of Egr1 and JunB. Collectively, our findings define an undocumented role of N-Ras in modulating of H3 acetylation and in gene regulation.

## 1. Introduction

Ras proteins play critical roles in a diverse range of biological responses including proliferation, differentiation, survival/apoptosis, and adhesion/motility [1]. Ras cycles between an active GTP-bound form and an inactive GDP-bound form, which is tightly regulated by guanine nucleotide exchange factors (GEFs) or GTPase-activating proteins (GAPs) [2]. Active GTP-bound Ras proteins interact with several effectors including Raf, Ral guanine nucleotide exchange factors (RalGEFs), and phosphatidylinositol 3-kinase (PI3K) that mediate the activation of transcription factors and cellular functions. Gain-of-function mutations in* Ras *genes are found in ~25% of all human cancers, with 98% mutations at G12, G13, or G61 [3], and are critical in tumor initiation and maintenance [4].

There are four Ras proteins—H-Ras, N-Ras, K-Ras 4A, and K-Ras 4B—with 80–90% amino acid sequence homology with major differences in the carboxyl termini [5]. Slight differences are found in the expression patterns of the four* Ras* genes according to the organ as well as during development and differentiation [6, 7]. Recently, it has been shown that each Ras isoform localizes to a distinct plasma membrane microdomain [8] and that Ras membrane orientation regulates effector utilization [3]. However, the specific function of each Ras isoform has not been elucidated.

Gene expression in eukaryotic cells is complex and tightly regulated at the level of chromatin dynamics and transcription factors. The distinct levels of chromatin architecture are dependent upon the higher-order structure of nucleosomes, which are composed of about 147 bp of DNA wrapped around an octamer of four core histones [9, 10]. The compact structure of chromatin is dynamically rearranged loosely or tightly during transcription, replication, and DNA repair by three major remodeling processes: histone modification, ATP-dependent chromatin remodeling, and histone variants exchange [11–13]. The altered chromatin structure can be categorized into two different types: euchromatin and heterochromatin. Euchromatin is found in the decondensed genomic regions and is mostly involved in gene activation by allowing transcription factors to bind DNA. Heterochromatin, on the other hand, appears highly compact and is transcriptionally inactive by blocking the access of transcription factors. Histone modification exemplifies an epigenetic mechanism that changes chromatin structure to affect transcriptional control. Histones can be modified at a specific amino acid with a diverse set of chemical modifications, such as acetylation, methylation, phosphorylation, and ubiquitylation. For example, acetylation on lysine residue neutralizes its positive charge and weakens the binding between the histone and the negatively charged DNA, hereby leading to the exposure of the DNA to regulatory proteins [14, 15]. In general, hyperacetylation of histone H3 and H4 is considered to be marks of gene activation, whereas hypoacetylation is linked to gene repression.

Recent studies revealed that deregulation of Ras signaling pathways contributes to aberrant histone modifications, leading to cancer development. For example, oncogenic H-Ras/PI3K signaling targets histone H3 acetylation at lysine 56 [16] and that oncogenic H/K-Ras alters the global and gene-specific histone modification pattern in colorectal carcinoma cells [17]. In addition, oncogenic H-Ras regulates CBP and Tip60, histone acetyltransferases, which modulate histone acetylation at local and global levels [18], and oncogenic H-Ras/Erk signaling axis increases histone 3 lysine 27 acetylation (H3K27ac) levels at enhancers near the transcription factors [19]. Although there is emerging evidence that histone modifications are highly implicated in Ras-mediated transformation, Ras isoform-specific signaling pathways associated with distinct histone modifications during cancer development are still poorly elucidated.

In this work, we identified oncogenic N-Ras as a critical activator of SRF-induced transcriptional activation by mediating downstream effector RGL2. We also found that oncogenic N-Ras signaling increases histone H3K9 and H3K23 acetylation at the chromatin level. Furthermore, N-Ras induced H3K9 acetylation (H3K9ac) is highly enriched in the loci of SRF target genes, leading to gene activation.

## 2. Materials and Methods

### 2.1. Materials

CV-1 and 293T cells were maintained in Dulbecco's modified Eagle's medium (DMEM) with 10% FBS and 1% penicillin/streptomycin in a 5% CO2 environment. Antibodies used were as follows: anti-3-Bromo-5′-deoxyuridine (BrdU) antibody was from GE healthcare; anti-HA-HRP antibody were from Roche; TRITC-conjugated anti-rat and FITC-conjugated anti-rabbit antibodies were from Jackson Immunoresearch; antibodies specific for H3K9ac, H3K18ac, H3K23ac, H3K9me3, and H3K36me3 were from active motif; antibodies for H3K4me3 and H3 were from Abcam. Anti-H3K27 antibody was from Millipore. Glutathione-Sepharose beads, BrdU, were from GE healthcare. SuperFect transfection reagent was obtained from Qiagen. Luciferase assay system was purchased from Promega. All other reagents were purchased from Sigma.

### 2.2. Plasmids

For construction of glutathione S-transferase- (GST-) fusions of H-Ras^G12V^, K-Ras^G12V^, and N-Ras^G12N^, PCR products were amplified with primers spanning the corresponding cDNA and subcloned into pGEX series vectors. Mammalian expression vectors for Ras isoforms were generated by inserting the corresponding cDNA fragments into pEGFP and pCGN-HA vectors. For reporter gene assay, PCR-amplified fragments of RalGDS-Ras binding domain (RBD) (residues 785–914) and RGL2-RBD (residues 643–777) were subcloned into pCMV-HA. SRE-Luc, NF-*κ*B-Luc, and AP-1-Luc reporter plasmids were purchased from Clontech.

### 2.3. Purification of Recombinant Ras Isoforms

GST-fused H-Ras^G12V^, K-Ras^G12V^, and N-Ras^G12N^ were expressed in* Escherichia coli, *BL-21 (DE3), and purified on glutathione-Sepharose beads as described previously [20]. For microinjection, GST-fused Ras proteins were dialyzed against microinjection buffer (20 mM Tris-acetate, pH 7.4, 20 mM NaCl, 1 mM MgCl_2_, 1 mM EDTA, and 5 mM 2-mercaptoethanol). Purified proteins were analyzed by 10% SDS-PAGE.

### 2.4. GTP Binding Activity

GST-fused Ras proteins (1.5 *μ*g) were incubated with the various concentrations of [^3^H]GTP (Amersham, 5.7 Ci/mmol, 1 *μ*Ci/ml) in assay buffer (50 mM Tris-HCl, pH 7.6, 50 mM NaCl, 5 mM MgCl_2_, 1 mM dithiolthreitol, and 10 mM EDTA) for 10 min at 30°C. After binding to glutathione beads, the beads were washed with cold washing buffer (50 mM Tris-HCl, pH 7.6, 50 mM NaCl, 5 mM MgCl_2_, and 10 mM EDTA) and were subject to liquid scintillating counting. Scatchard plot analysis was performed with linear regression using Harvard graphics.

### 2.5. Single Cell Microinjection and Immunostaining

To observe DNA synthesis by oncogenic Ras isoforms, the microinjection of recombinant Ras proteins has been described previously [21]. Briefly, CV-1 cells were grown on 12 mm glass coverslips for 24 hr and serum-starved for 24 hr. Cells were microinjected by using glass capillary needles made with a vertical pipette puller (David Kopf Instruments). 2 hr after microinjection, injected cells were treated with BrdU and further incubated for 16 hr at 37°C. After fixed with 3.7% formaldehyde, cells were subsequently incubated with rat anti-BrdU antibody, TRITC-conjugated anti-rat antibody, and FITC-conjugated anti-rabbit IgG antibody. In injection experiments, results represent the mean of at least three independent experiments in which at least 100 cells were injected. To observe cellular localization, CV-1 cells were grown on 12-mm glass coverslips and cotransfected with HA-H-Ras^G12V^ and GFP-K-Ras^G12V^ expression vector or HA-H-Ras^G12V^ and GFP-N-Ras^G12N^ expression vector for 24 hr. After incubation with serum-free DMEM for 20 hr, cells were fixed, subjected to immunostaining with anti-HA antibody, and imaged with confocal microscope (Carl Zeiss).

### 2.6. Reporter Gene Assay

CV-1 cells were grown and transiently transfected with reporter gene (100 ng) and expression vectors for oncogenic Ras isoforms along with RalGDS-RBD or RGL2-RBD for 24 hr. Cells were incubated in serum-free DMEM for 20 hr and lysed with reporter lysis buffer and assayed for luciferase activity using luciferase assay system (Promega).

### 2.7. Chromatin Extraction and Western Blot Analysis

Chromatin was extracted as described previously [22]. Briefly, cells were lysed in buffer A (10 mM HEPES, pH 7.4, 10 mM KCl, 1.5 mM MgCl_2_, 0.34 M sucrose, 10% glycerol, 1 mM DTT, 5 mM *β*-glycerophosphate, 10 mM NaF, protease inhibitor, and 0.2% TritonX-100). Nuclei were isolated by centrifugation (1,300 g for 10 min at 4°C), and the resulting nuclei pellet was resuspended in buffer B (3 mM EDTA, 0.2 mM EGTA, 1 mM DTT, 5 mM *β*-glycerophosphate, 10 mM NaF, and protease inhibitor). Chromatin pellet was washed three times with buffer B prior to sonication in Laemmli buffer.

### 2.8. Quantitative Reverse Transcription PCR (qRT-PCR) and Chromatin Immunoprecipitation (ChIP)

For qRT-PCR, total RNA was prepared using the TRIzol reagent (Invitrogen) and cDNA was prepared using M-MLV reverse transcriptase (Promega) according to the manufacturer's instructions. Real-time PCR was performed using the IQ SYBR Green Supermix and the IQ5 real-time cycler (Bio-Rad). Relative mRNA levels were normalized to *β*-actin mRNA levels. All reactions were run in triplicate, and the data presented are the average of three independent experiments. The following primers were used to quantify target gene expression:* Cyr6* (5′-CTCCCTGTTTTTGGAATGGA-3′ and 5′-TGGTCTTGCTGCATTTCTTG-3′),* Egr-1* (5′-TGACCGCAGAGTCTTTTCCT-3′ and reverse 5′-AGGCACAAGGGTACAAGACAGT-3′),* JunB* (5′-TGGAACAGCCCTTCTACCAC-3′ and 5′-GAAGAGGCGAGCTTGAGAGA-3′),* Scyl1* (5′-CTCCTCACTCACCTCCAAGC-3′and TGTCCTCTGCTGTGTCCTTG),* E2F5* (5′-CTGGAGGTACCCATTCCAGA-3′ and TGTTGCTCAGGCAGATTTTG-3′),* Npm1* (5′-AAAAAGCGCCAGTGAAGAAA-3′ and 5′-ACTTCCTCCACTGCCAGAGA-3′), and *β*-actin (5′-GTGGGGCGCCCCAGGCACCA-3′ and 5′-CTCCTTAATGTCACGCACGATTTC-3′). For ChIP assays, cells were fixed with 1% formaldehyde and processed for immunoprecipitation using antibodies against H3 and H3K9ac as recently described [23]. The purified DNA and input genomic DNA were analyzed by quantitative real-time PCR. Quantitative PCR analysis of the ChIP sample was normalized to the input genomic DNA. Primers used for quantitative real-time PCR are as follows:* Egr-1 (−0.4 kb)* (5′-GCGACCCGGAAATGCCATAT-3′ and 5′-CCTTCTTCCCTCCTCCCAGA-3′),* Egr-1 (+0.4 kb)* (5′-CCCACCATGGACAACTAC CC-3′ and 5′-CCTGAGGGTTGAAGGTGCTG-3′),* JunB (−0.3 kb)* (5′-GCACATACTGGGACCCTCAC-3′ and 5′-TGAGTGAGGGGTTTCAGGGA-3′),* JunB (+0.4 kb)* (5′- AACTCCTGAAACCGAGCCTG-3′ and 5′- CGAGCCCTGACC AGAAAAGT-3′),* E2F5 (+04 kb)* (5′-GGGCTGCTCACTACCAAGTT-3′ and 5′-CTACACCACGCCGCTAGAC-3′), and* Npm1 (+0.2 kb)* (5′-TTTTGGCCCCCAAGTTACGT-3′ and 5′-TACCCCAAAGTTCAGGTGCC-3′).

## 3. Result

### 3.1. Oncogenic Ras Isoforms Differentially Increase DNA Synthesis in CV-1 Cells

**Figure 1:**
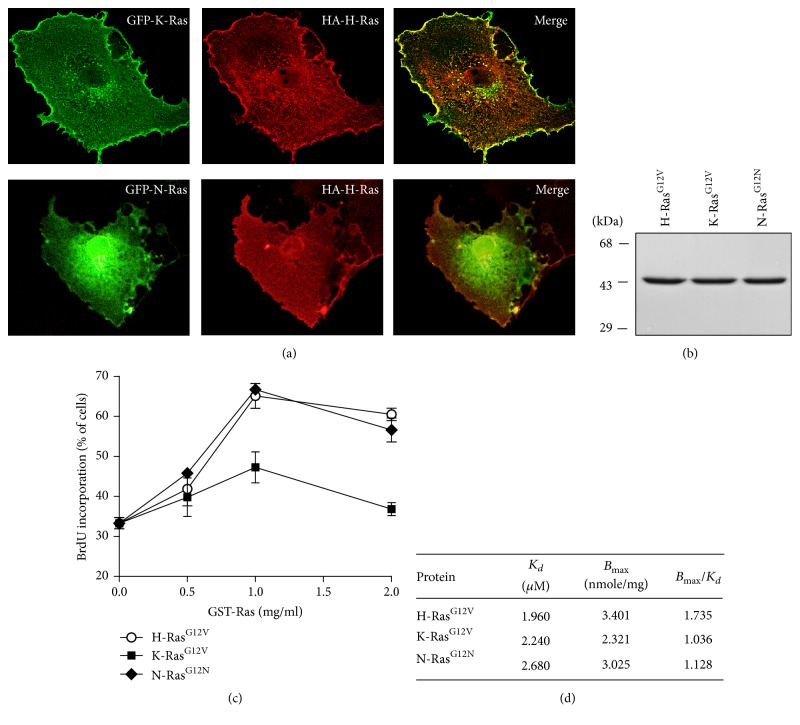
Oncogenic Ras isoforms differentially induce DNA synthesis in CV-1 cells. (a) CV-1 cells were cotransfected with HA-H-Ras^G12V^ and GFP-K-Ras^G12V^ expression vector or HA-H-Ras^G12V^ and GFP-N-Ras^G12N^ expression vector. Cells were fixed and subjected to immunostaining with anti-HA antibody. Representative images of single confocal section were presented. Scale bar, 10 *μ*m. (b) Recombinant Ras proteins were purified as described in Materials and Methods. The purity of the purified Ras proteins was analyzed by SDS-PAGE and Coomassie blue staining. (c) Serum-starved CV-1 cells were microinjected with GST-Ras (0.5, 1 or 2 mg/ml) in combination with rabbit IgG. The cells were incubated with BrdU for 16 hr. DNA synthesis in the injected cells was determined by incubation with rat anti-BrdU antibody followed by TRITC-conjugated anti-rat antibody and FITC-conjugated anti-rabbit IgG antibody. The results were represented as means ± SD from three determinations with over 200 cells injected. (d) GTP binding activity of oncogenic H/K/N-Ras. GST-fusion Ras proteins (1.5 *μ*g) were incubated with the varying concentrations of [^3^H]-GTP. Scatchard plot analysis was performed with the linear regression performed with Harvard graphics.

In order to evaluate the differential functions of oncogenic Ras isoforms, we first investigated the subcellular localization of Ras isoforms. After cotransfection of oncogenic K-Ras or N-Ras with H-Ras into CV-1 cells, their subcellular localizations were examined by confocal microscopic analysis. As shown in Figure 1(a), Ras isoforms were widely distributed, but differentially localized within CV-1 cells. K-Ras^G12V^ and H-Ras^G12V^ predominantly localized to the plasma membrane and cytosol whereas N-Ras^G12N^ was largely found in the perinuclear region. The differential localization of Ras isoforms suggests that these isoforms have distinct functions.

To further understand the differential roles of oncogenic Ras isoforms, we examined the effect of these isoforms on DNA synthesis using the single cell microinjection technique. We first purified constitutively active GST-fusion Ras isoforms (Figure 1(b)) and then microinjected them into CV-1 monkey kidney cells. Two hours after microinjection, cells were labeled with BrdU for 16 hr, and DNA synthesis was analyzed by indirect immunofluorescence. At the point of 1 mg/ml of Ras protein injection, BrdU incorporations by H-Ras^G12V^, K-Ras^G12V^ and N-Ras^G12N^ were increased to 63%, 48%, and 67% of injected cells, respectively (Figure 1(c)). Although all Ras isoforms induced DNA synthesis in a dose-dependent manner, H-Ras^G12V^ and N-Ras^G12N^ induced DNA synthesis more than K-Ras^G12V^. Ras is a GTP binding protein that switches between an active state with a bound GTP and an inactive state with a bound GDP. To exclude the possibility that the differences of DNA synthesis among Ras isoforms may be attributed to different GTP binding activities, we examined the kinetics of [^3^H] GTP binding to Ras isoforms. To do this, GST-fused Ras isoforms were incubated with [^3^H] GTP; then GTP-bound active Ras was measured by liquid scintillation. As shown in Figure 1(d), kinetic analysis of three Ras isoforms affinity for GTP showed that those had similar Bmax/Kd values (1.735 for H-Ras^G12V^, 1.036 for K-Ras^G12V^, and 1.128 for N-Ras^G12N^), indicating no significant difference in GTP binding activities among Ras isoforms. These results suggest that the differential DNA synthesis activity of oncogenic Ras isoforms can be attributed to distinct downstream signaling pathways rather than differences in GTP binding activity.

### 3.2. N-Ras^G12N^ Stimulates SRE Activation via RGL2

**Figure 2:**
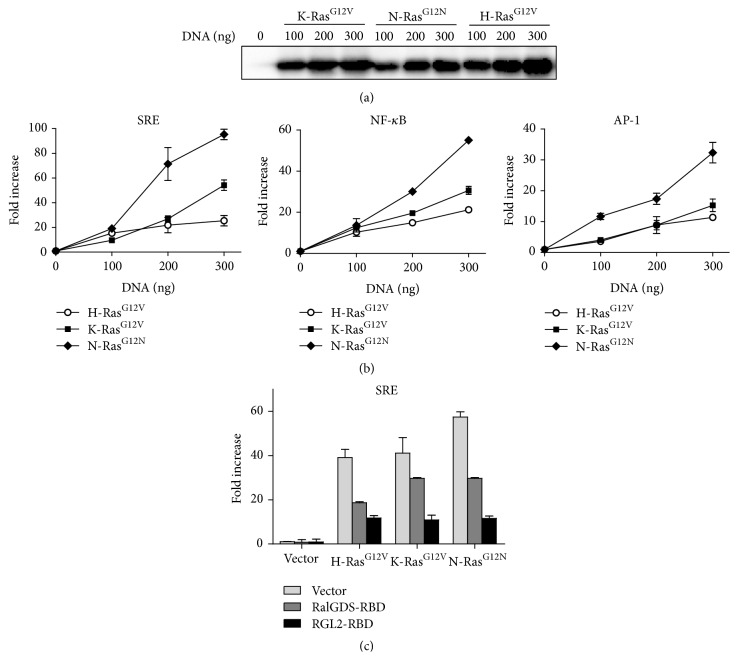
Oncogenic N-RasN12 stimulates SRE transactivation by mediating RGL2. (a) CV-1 cells were grown in a 12-well plate and transfected with the indicated reporter plasmids along with expression vectors for oncogenic H/K/N-Ras. Expression of oncogenic Ras isoforms was monitored by Western Blotting with anti-HA antibody. (b) The luciferase activity was determined 24 hr after transfection, and the relative activities of three independent experiments are shown as mean ± SD. (c) CV-1 cells were transfected with SRE-Luc reporter and expression vectors for oncogenic H/K/N-Ras together with mock, RalGDS-RBD, or RGL2-RBD plasmids as indicated. The luciferase activity was determined as shown in (b) and results show the mean ± SD of three experiments.

Oncogenic Ras isoforms differentially influence DNA synthesis by activating many transcription factors (e.g., AP-1, NF-kB, and SRF) that are involved in cell cycle progression [24–26]. In order to determine the role of oncogenic Ras isoforms in regulating transactivation of transcription factors, reporter gene assays were carried out on CV-1 cells transfected with a plasmid encoding oncogenic Ras isoform and a SRE-luciferase reporter construct. Interestingly, as shown in Figures 2(a) and 2(b), oncogenic N-Ras can more efficiently induce SRF-mediated transcriptional activation at least twofold compared to oncogenic H-Ras and K-Ras (hierarchy N-Ras^G12N^ > K-Ras^G12V^ > H-Ras^G12V^). In additional experiments with other reporter constructs (AP-1 and NF-kB), we found that oncogenic N-Ras is the most potent activator (Figure 2(b)). Given that oncogenic N-Ras pathway is highly associated with SRF-mediated transcription, we evaluated the downstream effector of oncogenic N-Ras. The family of GEFs for Ral (RalGDS, RGL1, and RLF/RGL2) have also been implicated as effector proteins for Ras [27]. Since it has been reported that the Ras-RalGDS axis is involved in c-fos gene expression [28], we investigated the role of RalGDS and RGL2 in N-Ras-induced SRE activation. To do this, we cotransfected pCGN-HA-Ras and pSRE-Luc with pCMV-HA-RalGDS-Ras binding domain (RBD) (785–914) or pCMV-HA-RGL2-RBD (643–777) and measured the luciferase activity. As shown in Figure 2(c), transfection of RalGDS-RBD similarly inhibited SRE activation by Ras isoforms (less than 50% inhibition). By contrast, RGL2-RBD significantly impaired SRE activation mediated by oncogenic Ras proteins. Particularly, N-Ras-mediated SRE activation was highly inhibited (80% inhibition), suggesting that N-RAS-RGL2 axis is a distinct pathway for SRE transactivation.

### 3.3. Oncogenic N-Ras Signaling Pathway Enhances Histone H3 Acetylation

**Figure 3:**
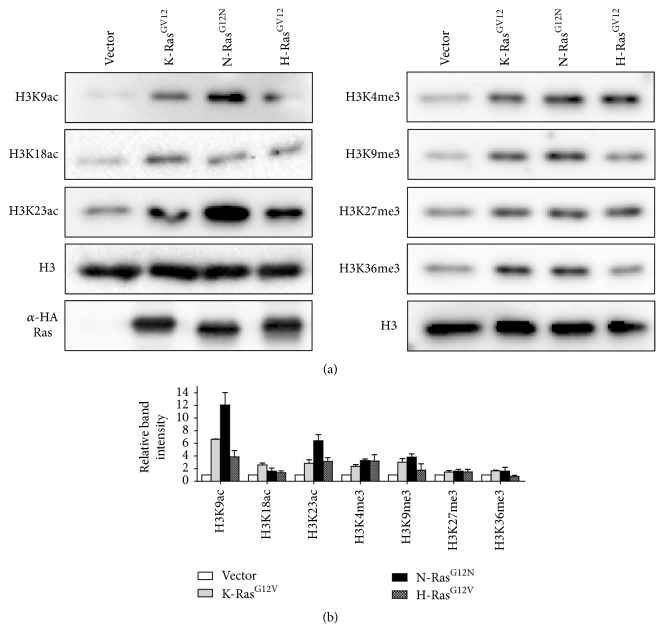
Oncogenic N-Ras induces histone H3 acetylation. (a) 293T cells were transfected with empty vector or the indicated oncogenic Ras isoform expressing vector. Chromatin was isolated and subjected to Western Blotting with the indicated antibodies. (b) Band intensities were quantitated by using ImageJ software, and the relative band intensities of three independent experiments are presented as mean ± SD. Values from cells transfected with empty vector are set to 1.

Ras mutations are frequently observed in many neoplastic cells [3]. Aberrant changes in histone modifications have been found in a variety of human cancers [29]. As oncogenic N-Ras is a more potent activator for SRE transactivation than other isoforms, we posited that three oncogenic Ras may differentially influence specific histone modifications. To this end, 293T cells were transiently transfected with a plasmid encoding HA-tagged K-Ras^G12V^, N-Ras^G12V^, or H-Ras^G12V^ and chromatin fractions were prepared from the transfected cells. As confirmed by immunoblotting, H3K9ac and H3K23ac showed an increase of 12-fold and 8-fold after the expression of oncogenic N-Ras, respectively (Figure 3). In contrast, transfection of two other Ras isoforms only slightly increased the level of H3K9ac/H3K23ac. We also observed no significant differences in H3 methylations as well as H3K18ac levels among the three Ras isoforms. These results provide robust evidence that oncogenic N-Ras signaling is predominantly linked to H3K9 and H3K23 acetylation levels.

### 3.4. H3K9 Acetylation by Oncogenic N-Ras Activates a Subset of SRF Target Genes

**Figure 4:**
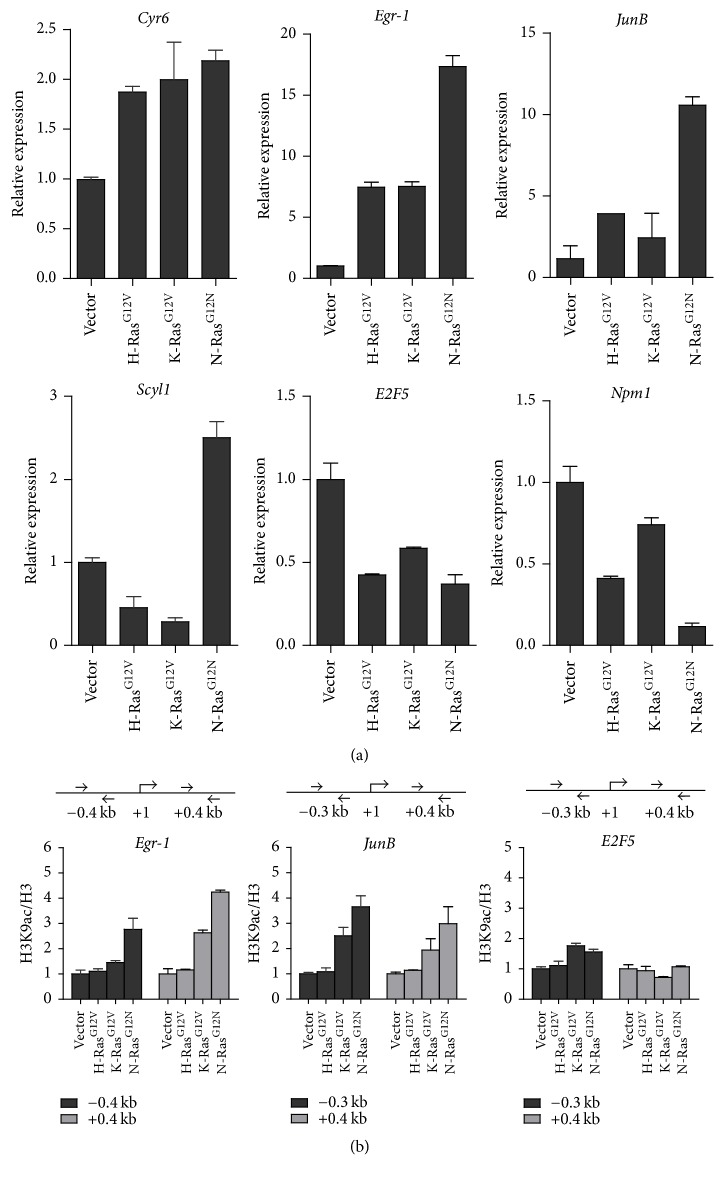
Oncogenic N-Ras-mediated H3 acetylation at lysine 9 is critical for target gene transcription. (a) 293T cells were transfected with the indicated expression plasmids for 48 hr. Total RNA was prepared from cells and qRT-PCR was performed using primers specific for* Cyr6*,* Egr-1, JunB, Scyl1, E2F5, and Npm1 *genes. The results shown are mean values from three independent experiments, and values derived from *β-Actin* in empty vector transfected cells are arbitrarily set to 1. (b) ChIP assays of* JunB* and* Egr-1* genes were performed using anti-H3K9ac and anti-H3 antibodies in 293T cells expressing Ras isoforms as in (a). ChIP-enriched DNA was determined for promoter and coding region by qPCR using the indicated primers. The data are the mean of three independent experiments ± SD.

Based on our findings that oncogenic N-Ras significantly enhances SRF-mediated transcription and H3 acetylation (Figures 2 and 3), we set out to investigate whether H3 acetylation by oncogenic N-Ras is necessary for activating SRF target genes. We first determined the expression levels of several SRF target genes using quantitative RT-PCR after transfection of Ras isoforms. Compared to H/K-Ras overexpression, transfection of N-Ras^G12N^ highly activated a subset of SRF target genes, including* Egr1* (17-fold increase)*, JunB *(11-fold increase), and* Scyl1 *(2.5-fold increase) (Figure 4(a)). In contrast, all three Ras isoforms resulted in no detectable differences in the gene expression of* Cyr6*,* E2F5,* and* Npm1*. These results indicate that the transcription of* Egr1, JunB,* and* Scyl1* genes is specifically regulated by N-Ras signaling. Next, we assessed the acetylation status of H3K9 and H3K23 at the* Egr1* and* JunB* genes by ChIP assays. Cross-linked chromatin was prepared from mock- or oncogenic H/K/N-Ras-transfected cells, and the precipitated DNA was amplified by real-time qPCR using primers specific for promoter region (−0.3 kb to −0.4 kb) and coding region (+0.4 kb). In agreement with our RT-PCR analysis, the enrichments of H3K9ac at both promoter and coding regions were significantly increased with oncogenic N-Ras transfection (3- to 4-fold increase), as compared to transfection of oncogenic K- or H-Ras transfection (less than 2-fold increase) (Figure 4(b)). However, there were no notable increases in H3K23ac levels at the* Egr1* and* JunB* loci (data not shown). Consistent with our RT-PCR analysis, H3K9ac was minimally detected in the* E2F5* gene when Ras isoforms were overexpressed (Figure 4(b)).

## 4. Discussion

Although Ras isoforms have a high degree of sequence homology, there is growing evidence that differences in functional properties of Ras isoforms may be implicated in cancer development [4, 6, 7]. However, the epigenetic mechanism underlying the link between Ras pathways and gene expression is largely unknown. In this study, we would suggest the distinct role of oncogenic N-Ras-RGL2 axis in inducing DNA synthesis as well as in transactivating a subset of SRF target genes via histone acetylation.

Our single cell microinjection studies demonstrate that oncogenic N-Ras has a significant increase in BrdU incorporation into DNA during DNA synthesis, thereby enhancing cell proliferation. Concomitant with microinjection data, reporter assays also reveal that oncogenic N-Ras highly transactivates a set of immediate early genes implicated in cell cycle progression. In contrast, transcriptional activity driven by both H-Ras and K-Ras was less than that of N-Ras. These results suggest that oncogenic N-Ras is a more potent transcriptional activator of early cell cycle progression genes than H-Ras and K-Ras.

The previous studies demonstrated that specific Ras isoforms preferentially interact with Ras effectors to trigger a variety of signaling pathways [30, 31]. For example, the members of Ral GEFs (RalGDS, RGL1, RGL2/Rlf, and RGL3) associate with active Ras proteins through Ras binding domain and activate RalA and RalB [32]. In this study, we observed that RGL2-RBD preferentially inhibits oncogenic N-Ras-derived SRE transactivation while RalGDS-RBD has less inhibitory effect on the SRE activation. Because Ras binding domains of RalGEFs have limited sequence homology (17%–33%) [33], those sequence differences may play an important role in distinct Ras downstream signaling pathways. However, additional studies are needed to understand the specific mechanisms underlying the interaction between N-Ras and RGL2.

Recent studies have indicated that oncogenic Ras is implicated in the alteration of chromatin structures and histone modifications [34]. Sánchez-Molina et al. have shown that oncogenic H-Ras-transformed fibroblast has an increase of global H4 acetylation, rendering more decondensed nucleosome architectures [18]. An important finding in our study was that oncogenic N-Ras significantly increases levels of H3K9ac and H3K23ac possibly through RGL2 signaling pathway. These histone modifications are well known as euchromatin marks and often correlated with active transcription [35]. Indeed, we found that oncogenic N-Ras upregulates a set of SRF target genes by enhancing H3K9ac levels at both promoter and coding regions of those genes. However, we did not detect obvious changes in H3K23ac levels on those genes. These results suggest that acetylation of H3K9 and H3K23 induced by N-Ras may be differentially required for transactivating target genes although oncogenic N-Ras increased the acetylation of H3K9 and H3K23 at a global level. Recent studies demonstrate that H-Ras signaling induced reduction in H3K56ac levels by mediating PI3K, thereby causing transcriptional activation [16]. In agreement with our findings, oncogenic H-Ras overexpression did not significantly affect other histone modifications including H3K9ac, H3K14ac, H3K18ac, and H4ac. Another study showed that H-Ras-induced signaling pathway MEK-ERK-MSK1 decreases global histone methylation at lysine 27, which is associated with gene repression [17]. Based on our observations, together with previous studies, we propose that oncogenic Ras isoforms differentially regulate histone modifications through different downstream signaling pathways. Thus, it will be interesting to examine how Ras isoform-specific differences in downstream signaling pathways could contribute to the change of histone modifications.

Several questions have arisen from our study. How can oncogenic N-Ras-RGL2 axis enhance histone H3 acetylation? Which histone acetyltransferases (HATs) are linked to this pathway? Considering the previous report that oncogenic H-Ras regulates CBP and Tip60, histone acetyltransferases, affecting histone acetylations [18], it is possible that those HATs could be one of the downstream effectors for N-Ras pathway. Hence, such questions will be the aims for future studies.

Taken together, we demonstrated that oncogenic N-Ras stimulate SRF target genes by modulating H3K9ac levels. We found that H3K9ac is highly enriched at the promoter and coding regions of target genes, thereby influencing cancer cell development. This mechanism may provide a novel therapeutic target for cancer treatment.
